# Universal slip detection of robotic hand with tactile sensing

**DOI:** 10.3389/fnbot.2025.1478758

**Published:** 2025-02-05

**Authors:** Chuangri Zhao, Yang Yu, Zeqi Ye, Ziyang Tian, Yifan Zhang, Ling-Li Zeng

**Affiliations:** College of Intelligence Science and Technology, National University of Defense Technology, Changsha, China

**Keywords:** slip detection, five-finger robotic hand, deep learning, 3-axial force tactile sensor, grasp types

## Abstract

Slip detection is to recognize whether an object remains stable during grasping, which can significantly enhance manipulation dexterity. In this study, we explore slip detection for five-finger robotic hands being capable of performing various grasp types, and detect slippage across all five fingers as a whole rather than concentrating on individual fingertips. First, we constructed a dataset collected during the grasping of common objects from daily life across six grasp types, comprising more than 200 k data points. Second, according to the principle of deep double descent, we designed a lightweight universal slip detection convolutional network for different grasp types (USDConvNet-DG) to classify grasp states (no-touch, slipping, and stable grasp). By combining frequency with time domain features, the network achieves a computation time of only 1.26 ms and an average accuracy of over 97% on both the validation and test datasets, demonstrating strong generalization capabilities. Furthermore, we validated the proposed USDConvNet-DG in real-time grasp force adjustment in real-world scenarios, showing that it can effectively improve the stability and reliability of robotic manipulation.

## 1 Introduction

The importance of tactile feedback has been emphasized by studies in human motor control, which show that stable object manipulation is difficult without this sensory input (Johansson and Vallbo, 1979). Tactile perception plays a crucial role in human object grasping. When slippage occurs, humans can promptly adjust their grip force and strategy to prevent the object from falling. This ability significantly enhances the flexibility and stability of object manipulation by the human hand (Johansson and Flanagan, 2009).

With the increasing application of robots in unstructured environments, they are required to perform more flexible manipulation tasks and achieve stable grasping, similar to humans (Chen et al., 2018). Although the accuracy and resolution of artificial tactile sensors still fall short of human tactile capabilities, they still play a significant role in improving grasping stability in robotic systems (Grover et al., 2022). They provide essential information about the interaction between the hand and the object, enabling quicker and more accurate slip detection than vision-based methods alone (Johansson and Westling, 1984; Westling and Johansson, 1984). Robots equipped with reliable tactile sensing can significantly improve their dexterous manipulation capabilities and achieve stable grasping of common objects (Cui et al., 2020). One of the most important dexterous robot manipulation tasks using the sense of touch is to detect or predict sliding while grasping a manipulated object. Slip detection is essential for ensuring stable robotic grasping, which is crucial for preventing objects from slipping or falling during manipulation. Detecting slip allows robotic systems to adjust their grasp strategies and forces in real-time, ensuring that objects remain securely held (Yan et al., 2022; James and Lepora, 2021).

However, there are still some challenges. On the one hand, as sensor arrays become increasingly dense and sensing dimensions expand, traditional methods struggle to construct suitable models for detecting slippage. On the other hand, while previous research has made notable progress in slip detection for two\three-fingered robotic grippers (Chen et al., 2018; Romeo and Zollo, 2020), slip detection for five-fingered dexterous hands presents unique challenges because the complexity of grasp types that five-fingered hands can perform, as well as the need for algorithms that can generalize across a variety of object shapes, sizes, and materials.

**Figure 2:**
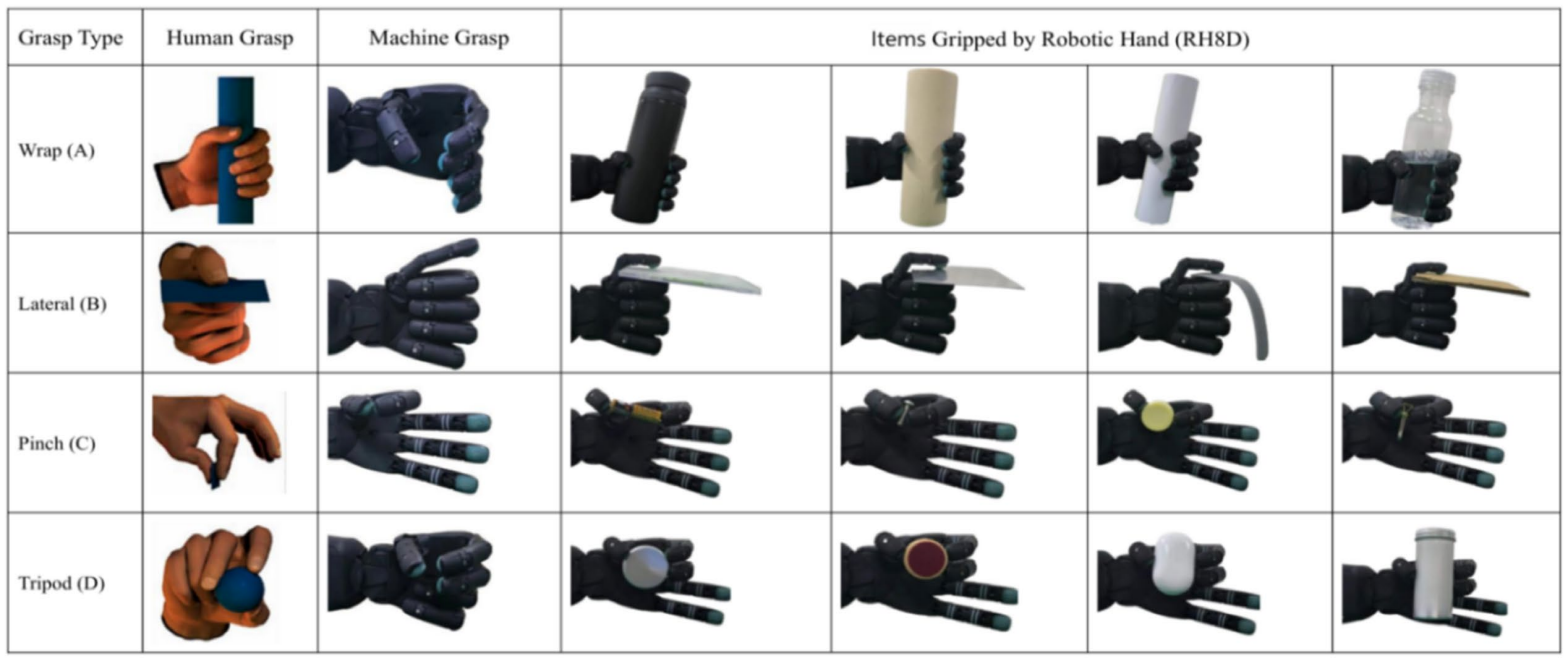
Grasp types and items for training the model. The first column represents the grasp type names. The second column indicates the grasping type for a human hand. The third column represents the corresponding grasping posture for the robotic hand. Columns 4, 5, 6, and 7 depict the items grasped for the corresponding grasp types.

In this study, we present a solution to the problem of slip detection in five-fingered robotic hands Five-finger robotic hand can perform a wide range of grasp types, each with unique contact dynamics, making it challenging to develop a one-size-fits-all solution. To address this challenge, we propose a Universal Slip Detection Framework for Different Grasp Types (USDFrame-DG), designed to handle the complexities associated with various grasp types and object properties. In summary, the main contributions of this work are as follows:

According to the reference document (Feix et al., 2016), six common and significantly different grasp types were selected, as shown in Figure 1. A large amount of grasp state data (no-touch, slip, no-slip) was collected during these six grasp types. The 16 objects used for grasping, as shown in Figure 2, are made from materials commonly found in daily life, such as plastic, steel, and wood.
**Figure 1:**
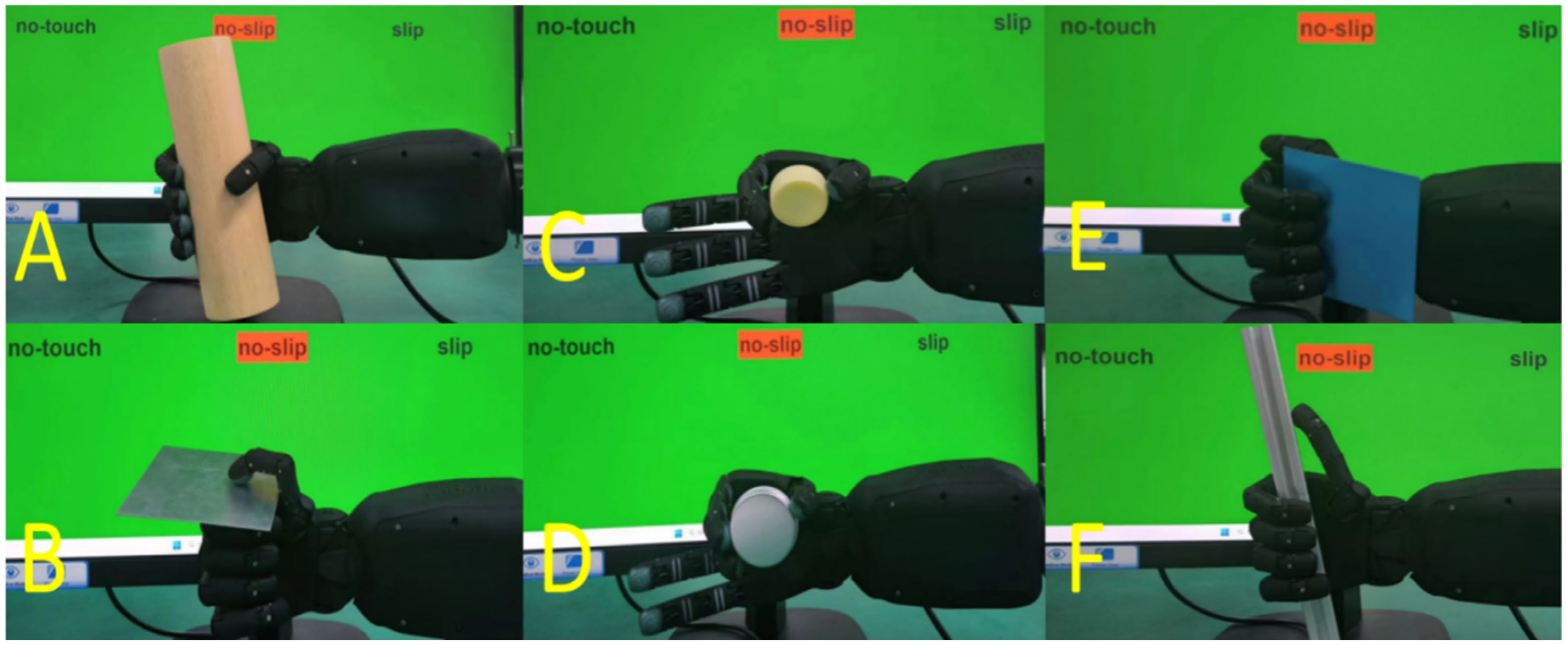
Grasp types and grasp state visualization. USDConvNet-DG trained for application in real-world scenarios.

A novel universal slip detection framework (USDFrame-DG) was proposed, focusing on efficiently collecting large-scale datasets and combing the frequency with time domains to achieve improved recognition performance.
To validate which network architecture is better suited to address this problem, we compared four classic classification methods: Support Vector Machine (SVM), Long Short-Term Memory (LSTM) network, Residual Neural Network (ResNet), and Transformer. According to the results of the comparison, a lightweight and efficient USDConvNet-DG was designed, achieving more than 97% accuracy on both the validation and test sets. This capability highlights the universality and generalization of the proposed framework.
We evaluated the performance of different methods, the contribution of various grasp types, and the performance of USDConvNet-DG trained with different numbers of grasp types. Additionally, we developed a physical demonstration system to showcase network’s ability to detect slip in real-time, as shown in Figure 1. Furthermore, we increased the object’s weight after achieving a stable grasp to verify whether the system can adjust the grasping force in real-time. Video demonstrations have been uploaded to the GitHub repository and are available at https://github.com/sunshine486/show.

## 2 Related works

Existing methods for detecting slippage during grasping can be divided into two categories: (1) analysis-based methods and (2) learning-based methods. Analysis-based methods typically identify grasp states using two key features: frequency and friction. Learning-based methods usually involve collecting data on slip and no-slip states to train a classification model.

These are some representative works based on changes in friction force. The first, proposed by Claudio Melchiorri, detects slippage by comparing the ratio of friction force to grasp force with the coefficient of friction (Melchiorri, 2000). The second, introduced by Beccai et al. (2008), utilizes friction cones to achieve slip detection, but with a delay exceeding 20 ms both methods operate on similar principles. Another approach, proposed by Song and Liu, employs the Break-Away Friction Ratio (BF-ratio) to predict slippage during the grasping process (Song et al., 2013). Although this method completes the prediction within just 4.2 ms, it requires 5–7 s to determine the friction coefficient through haptic surface exploration and has been validated in only three scenarios.

The signal spectra of slipping and non-slipping states are significantly different (Zhang et al., 2016). Specifically, when the grasp is stable, the signal primarily consists of low-frequency components; however, during slipping, the signal shifts to higher frequencies. Holweg et al. (1996) noted that the normal forces measured by the tactile sensor fluctuate at a certain frequency during slip due to the elasticity of rubber. Techniques such as Discrete Wavelet Transform (DWT) (Shensa, 1992) and Fast Fourier Transform (FFT) (Duhamel and Vetterli, 1990) have been employed to detect slip vibrations during robotic grasping. DWT is typically used for filtering, followed by a manually defined threshold to distinguish between slip and no-slip states (Zhang et al., 2016; Deng et al., 2017), making it more suitable for analysis-based methods. Zeng et al. (2022) utilized Discrete Wavelet Transform (DWT) to extract high-frequency signals, which were then compared against predefined thresholds to achieve slip detection. Similarly, Yang and Wu (2021) divided the slipping process into two phases: the initial slip phase and the slip suppression phase, with detection thresholds estimated separately for each phase. Both studies were conducted using a prosthetic hand. It is worth noting that Romeo et al. achieved slip detection at the hardware level using filters and on–off circuits (Romeo et al., 2021), which provides higher integration. However, adjusting thresholds and filters requires replacing components such as inductors and capacitors, making it challenging for non-technical users.

Analysis-based methods for slip detection generally rely on single touch areas, which overlook the spatial characteristics of different fingertips and the variations in touch areas caused by different grasp types. The slip detection performance of these methods is highly dependent on specific touch conditions. Consequently, parameters such as thresholds and filters lack generalization when applied to new contact scenarios introduced by a wide range of objects (Cui et al., 2024). Moreover, manually setting these parameters is time-consuming and cumbersome, requiring a certain level of engineering expertise.

In learning-based methods, slip detection is commonly formulated as a binary classification problem (slip/non-slip). With the rapid advancements in machine learning and the growing diversity of tactile sensors, machine learning techniques have been increasingly applied to slip detection, resulting in impressive outcomes.

In the field of machine-learning-based slip detection, the work of James and Lepora (2021) is particularly noteworthy. They utilized a sensor array to calculate the rate of change of pin positions per frame and compared three distinct binary classifiers (Threshold Classifier, SVMs, and Logistic Regression), achieving promising results in real-world scenarios.

In previous research, most studies are based on two-finger grippers and use LSTM network. Zhang et al. (2018) developed a novel optical-based tactile sensor (FingerVision), and proposed a sliding classification framework based on ConvLSTM (Convolutional Long Short-Term Memory) networks. Begalinova et al. (2022) employed an LSTM model trained on low-cost tactile sensors and evaluated the model using a two-finger gripper. Xie et al. (2023) employs LSTM networks for sliding detection and found that robotic grasping with slip detection has a success rate nearly 15% higher than grasping without slip detection. Fiedler et al. (2023) utilizes sliding detection based on a two-finger gripper to achieve grasping of textile objects. Yan et al. (2022) employed multimodal machine learning, combining visual and tactile information using a convolutional neural network-temporal convolutional neural network (CNN-TCN), achieving a detection accuracy of 88.7% for sliding detection with a two-finger gripper. James et al. (2018) used the TacTip sensor and Support Vector Machine (SVM) algorithm to classify sliding and stationary states, achieving an accuracy of 99.88%. However, this result was obtained only in structured environments, and the actual performance was not tested.

In addition, there are some studies based on five-finger robotic hands, but they have only achieved slip detection for a single grasp type. Zapata-Impata et al. (2019) utilized ConvLSTM to detect the direction of object sliding on the fingertip. The sensors used in the papers are BioTac, which is very expensive. Mi et al. (2021) propose two novel methods based on Graph Convolution Network (GCN) for robotic stability classification. Grover et al. (2022) train a temporal convolution neural network (TCN) to detect slip that achieves an accuracy of over 91% on average on validation dataset. These two methods are based on three-finger grippers. Garcia-Garcia et al. (2019) constructed a graph neural network to predict the stability of grasping, but their work was based on three fingers. Deng et al. (2020) utilized sliding detection based on LSTM networks as feedback to control the grasping force.

The above studies demonstrate the effectiveness and robustness of learning-based slip detection methods utilizing tactile sensing. However, there remain several challenges in this field, as outlined below:

1. Traditional analysis-based methods require manual adjustment of thresholds and filters, which is not only time-consuming and cumbersome but also demands a certain level of engineering expertise.
2. Tactile sensors are becoming denser arrays, capable of perceiving multi-dimensional forces and more diverse sensing modalities. Analysis-based methods struggle to construct suitable mathematical models to handle this complexity.
3. Previous learning-based studies have primarily focused on grippers or two−/three-finger robotic hand platforms, which are limited to a single grasping style. In contrast, five-finger dexterous hands are capable of performing a wide range of grasp types, making slip detection significantly more complex. As shown in Table 1, models trained solely on state data from a single grasp type exhibit poor performance in detecting slips for other grasp types, indicating a lack of generalization capability.
4. Slip detection for five-finger robotic hands usually detect slippage in individual fingertip regions. This study treats across all five fingers as a whole for slip detection. However, this approach lacks sufficient datasets and requires further exploration of suitable network architectures.

**Table 1:** Accuracy of USDConvNet-DG trained with varying numbers of grasp types.

Grasp types	1	2	3	4
Training dataset	A, B, C, D	AB, AC, AD, BC, BD, CD	ABC, ABD, ADC, BCD	ABCD
Accuracy	45.1 ± 6.12%	80.7 ± 6.43%	93.7 ± 2.92%	95.7 ± 2.41%

In this study, we focus on universal slip detection for different grasp types. Inspired by prior work and integrating analysis-based and learning-based methods, we propose a novel slip detection framework and network.

## 3 Method

**Figure 3:**
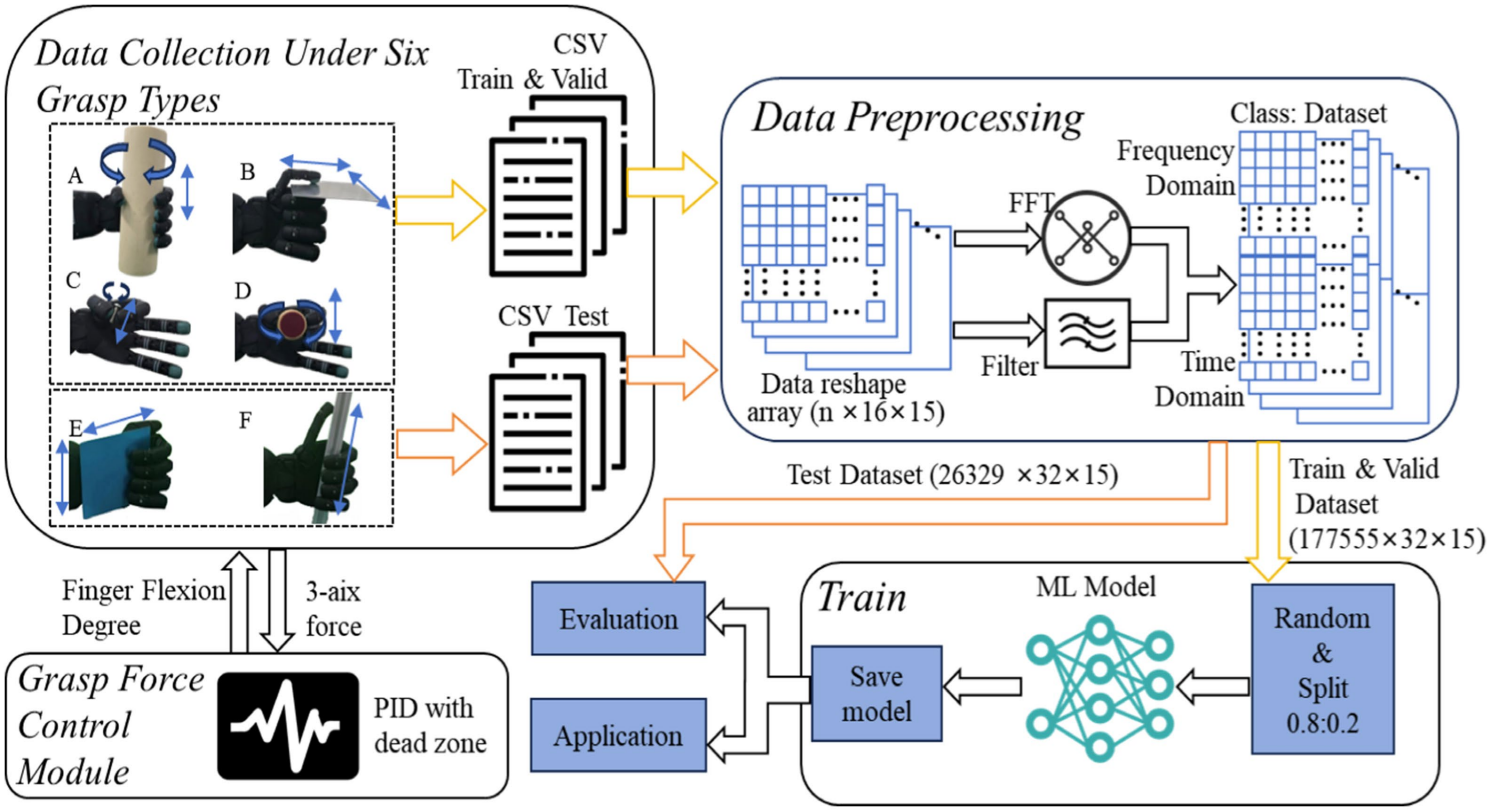
USD Frame-DG: universal slip detection framework for different grasp types.

To achieve universal slip detection across different grasp types, we propose a general slip detection framework: USDFrame-DG, as shown in Figure 3. The framework consists of four key components: Grasp Force Control Module, Data Collection for Six Grasp Types, Data Preprocessing, and Model Training, each of which will be detailed below. Over 200 k data samples covering slipping, stable grasping, and non-touch states were collected to train the models. The dataset for slipping and stable grasping states was collected using various grasp types and everyday objects, ensuring the model’s applicability to real-world scenarios.

### 3.1 Hardware setting

**Figure 4:**
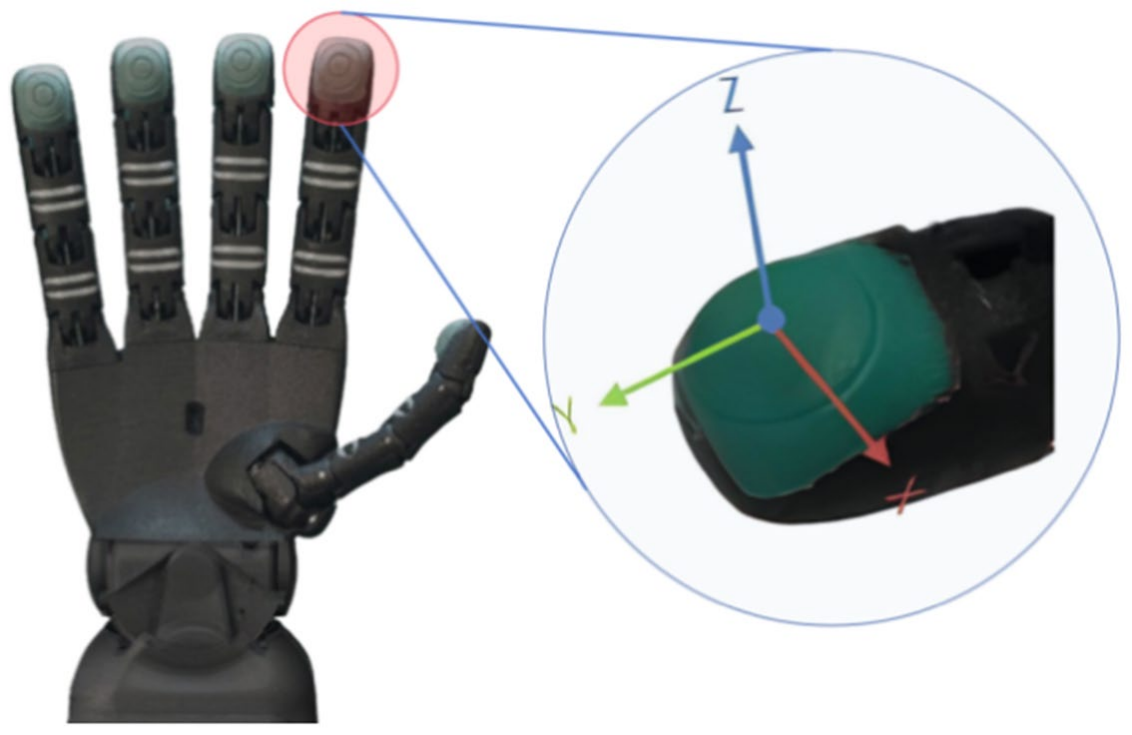
RH8D adult robot hand and FTS-3.

The model of five-finger robotic hand used in our experiments is RH8D, designed by Seed Robotics, as shown in Figure 4. Inspired by the human hand, it is capable of performing essential grasp types, featuring tendon-driven mechanisms and underactuated design. The RH8D can be mounted at the end of a six-degree-of-freedom robotic arm and features 19 degrees of freedom, including an opposable thumb and a full spherical wrist joint. It’s three-segment fingers are powered by smart actuators housed entirely within the unit, offering payload capabilities (750 g in 3D space and 2.5 kg vertical pull). Inspired by the human hand, the RH8D provides advanced sensing and data acquisition on all actuated joints, including real-time feedback on position, speed, current, and PWM output. Additional features include a palm Time of Flight (ToF) distance sensor, optional capacitive touchpads for enhanced human-robot interaction, and reinforced design elements like Dyneema tendons and magnetic detachment for durability.

The Fingertips Tactile Sensors (FTS) used in this study are 3-axis force sensors designed for precise force measurement, as shown in Figure 4. These sensors measure forces along the X, Y, and Z axes and are optimized for forces in the 0–10 N range, offering a resolution of 1mN. For higher forces (10–30 N), an extended range model is used, with a resolution of 10mN in this range. The sensors operate with a sampling frequency of 50 Hz and have an overload capability of up to 50 N. Additionally, there is a 20mN offset when the sensors do not touch objects. The FTS works via an array of MEMS (Micro-Electromechanical System) sensors, which are highly resistant to magnetic field interference. Noise levels are minimal (on the order of millinewtons), making the sensors well-suited for practical applications. The sensors are pre-calibrated and exhibit linear performance in the typical force range of 10°–30° and beyond. While fast temperature changes may cause slight drift (up to 100mN in extreme conditions), these effects are generally negligible in most scenarios. For more technical details on the sensor specifications and design, we refer readers to the Seed Robotics documentation and related resources.

### 3.2 Grasp types

The five-finger robotic hand offers a higher degree of freedom compared to two-finger and three-finger grippers, allowing it to generate many more grasp types. Feix et al. (2016) summarized 33 common grasp types used by humans, which can be grouped into six categories. When considering only hand configuration, without taking into account object shape or size, these 33 grasp types can be reduced to 17 more general types. Although the RH8D features 19 degrees of freedom, human hands possess 27 degrees of freedom (Agur and Dalley II, 2023), meaning the RH8D is unable to perform all the grasp types like human.

We initially collected slip and non-slip data for one grasp type and used this data to train a USDConvNet-DG model. The recognition accuracy exceeded 96% for the trained grasp type (A), but dropped below 70% for another grasp type (E). Although the model showed some generalization ability, its accuracy was insufficient for adjusting grasp force and strategy. Surprisingly, we discovered that it wasn’t necessary to collect sliding data for all 33 grasp types. By gathering data for a few significantly different grasp types, the model could generalize effectively to other grasp types.

In the end, we selected four significantly different grasp types that the robotic hand could perform, as shown in Figure 2. These four types are suited for various scenarios: “Wrap (A)” for grasping long and large objects, “Lateral (B)” for flat objects, “Pinch (C)” for small and delicate objects, and “Tripod (D)” for smaller objects. The remaining two grasp types (E and F) are used to test generalization.

In our experiment, we chose 16 common items to collect grasping data, as shown in Figure 2. The weight of these objects ranged from 10 g to 300 g, and the materials included plastic, metal, wood, paper, and other commonly encountered substances.

### 3.3 Grasp force control

**Figure 5:**
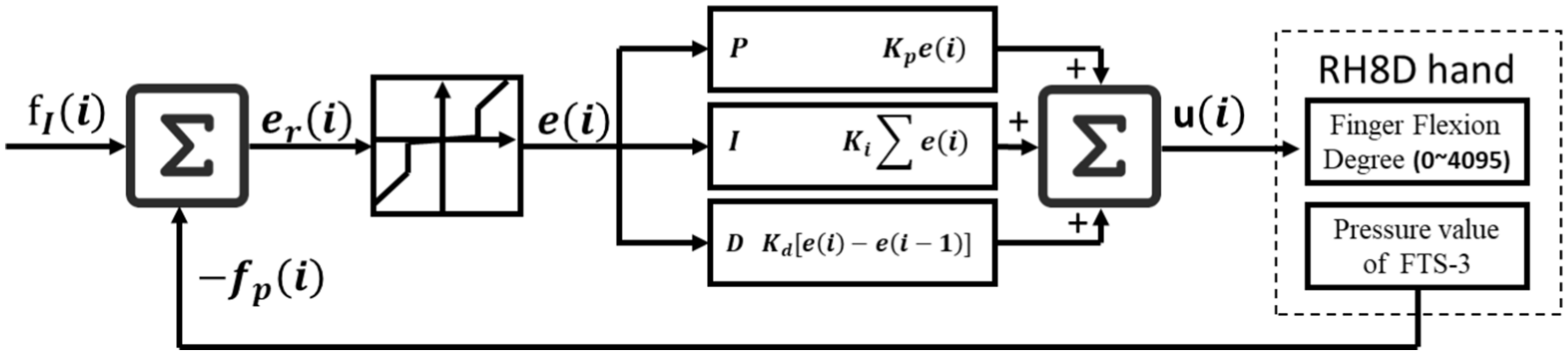
Grasp force control module.

A PID (Proportional Integral Derivative) controller with a dead zone is used to control the robotic hand’s grasping force. The grasping force of each finger can be controlled individually. As shown in the Figure 5. $f_{I}\left(i\right)$ represents the aim grasping force, and $f_{p}\left(i\right)$ represents the synthesis of the three-directional force detected by the FTS, calculated as follows:

$$f_{p}\left(i\right)=\sqrt{f_{x}^{2}+f_{y}^{2}+f_{z}^{2}}$$

The difference between $f_{I}\left(i\right)$ and $f_{p}\left(i\right)$ is denoted as $e_{r}\left(i\right)$ . To prevent oscillation of the robotic hand during grasping, the range of change in $e_{r}\left(i\right)$ needs to be limited, as shown in the following formula. After multiple tests, setting the threshold *𝑡ℎ𝑟* to 150mN was the most suitable.

$$e\left(i\right)=\{\begin{array}{c} 0.01e_{r}\left(i\right)\;\mathrm{if}\;e_{r}\left(i\right)<thr\\ e_{r}\left(i\right)\;\mathrm{else}\; \end{array}$$

The value 𝑢 is obtained from the PID controller, which represents the flexion degree of each finger (range: 0–4,095). The formula is as follows:

$$u\left(i\right)=K_{p}e\left(i\right)+K_{i}\sum e\left(i\right)+K_{d}\left[e\left(i\right)-e\left(i-1\right)\right]$$

After extensive testing, the most suitable values for $K_{p}$ , $K_{i}$ and $K_{d}$ were found to be 0.4, 0.04, and 0.5, respectively.

### 3.4 Data collection

In this work, slip detection is treated as a classification problem with three categories: no-touch, no-slip, and slipping. Deep learning methods rely on large amounts of data. It is easy to collect data for the no-touch and no-slip states, but collecting enough data for the slipping state is challenging because it occurs in an instant. Data from the FTS is directly saved as no-touch when no object is being grasped. During stable grasps of six types, the collected data belongs to the no-slip category. We tried two methods to deal with the challenge of collecting slipping data.

The first method involves slowly pulling out the object after the robotic hand has stably grasped it. Approximately 2000 data points can be collected within 5 s when the sampling rate is set to 50 Hz. Although training a network with this data results in high accuracy on the validation set, its performance on the test set and in real-world applications is poor. Through continuous reflection and analysis, we found that the abrupt change in force during slipping is the key feature.

To capture this feature, we proposed another data collection method: after the robotic hand grasps the object, an external force is applied by hand to move the object back and forth quickly in the direction shown in Figure 1. This allows for the rapid collection of a large amount of slipping data, making it possible to use neural network-based classification methods. The final test results demonstrated significant improvements. We believe that this method can also be used to quickly collect a large amount of effective slipping data for robotic hands equipped with other types of sensors. For grasp types A, B, C, and D, the data is used for training and validation, while grasp types E and F are used for testing to evaluate the generalization of the detection model.

### 3.5 Data preprocessing

Each data record comprises 15 measurements, corresponding to the force components along three axes (X, Y, Z) for each of the five fingers. Noise removal from the dataset is manually performed, with particular attention given to the initial and final segments of the data sequences. To maintain sample balance, excessively long sequences are trimmed. Once processed, the data is ready to construct the training and test sets. The final dataset includes over 200,000 scalar data points sampled at a frequency of 50 Hz.

Since the collected data represents time series information with inherent periodicity and autocorrelation characteristics, training the model using a single data record results in suboptimal performance. Instead, combining multiple adjacent data records into a single sample is more effective, as it enables the system to observe force variations over a period of time, which is crucial for detecting slippage. However, using an excessively long observation period compromises real-time performance. After conducting extensive tests, we found that using a stride of 1 and combining 16 adjacent data records into a 16 × 15 array yields the best practical results. For example, if 2000 data points are collected in one session, the first 16 records form the first array, the second to the 17th records form the second array, the third to the 18th records form the third array, and so on, until the final 16 records form the last array. This structure also facilitates the application of FFT analysis.

In the collected slipping dataset, a small portion of noise is difficult to manually remove, which can significantly affect the trained model. A high-pass filter is used to preprocess the slipping data because the frequency of the slip signal is higher. The calculation formula is as follows:

$$y\left(i\right)=0.2x\left(i\right)-0.8y\left(i-1\right)$$

$x\left(i\right)$ represents the i -th array. By applying a filtering method, the model’s accuracy improved to a certain extent. Since an object generates vibrations during slipping, there is a distinctive spectral distribution in the frequency domain that can be used as a feature for training the model. A Fast Fourier Transform (FFT) is applied individually to each column of the data, resulting in a 16 × 15 matrix. This matrix is then combined with the filtered time-domain matrix, producing a 32 × 15 matrix where the first 16 rows represent the frequency domain, and the last 16 rows represent the time domain.

Labels in a one-hot format are assigned based on the grasping states: [1,0,0] for no-slip, [0,1,0] for slip, and [0,0,1] for no-touch. A total of 177,555 matrices (A, B, C, and D) are randomly divided into training and validation sets in an 80%:20% ratio, while 26,329 matrices (E and F) were reserved for testing, as shown in Table 2. The ratio of the three classes—no-touch, slip, and no-slip—is approximately 24%:38%:38%.

**Table 2:** Class distribution of grasp states across different grasp types.

Grasp type	A	B	C	D	E	F	Total
Slip	15,448	16,512	16,752	16,137	6,643	6,587	78,079
No-slip	15,948	15,538	15,987	16,392	6,513	6,586	76,964
No-touch	48,841						48,841

### 3.6 Network architecture

For slip detection, different types of sensors generate different data types, so there is no single model that fits all sensors. To address this, we designed four three-classification algorithms based on four classical models (SVM, LSTM, Residual Convolutional Neural Network and Transformer). These three categories are sliding, non-sliding, and no-touch. Below, we will describe these four models in detail.

**Figure 6:**
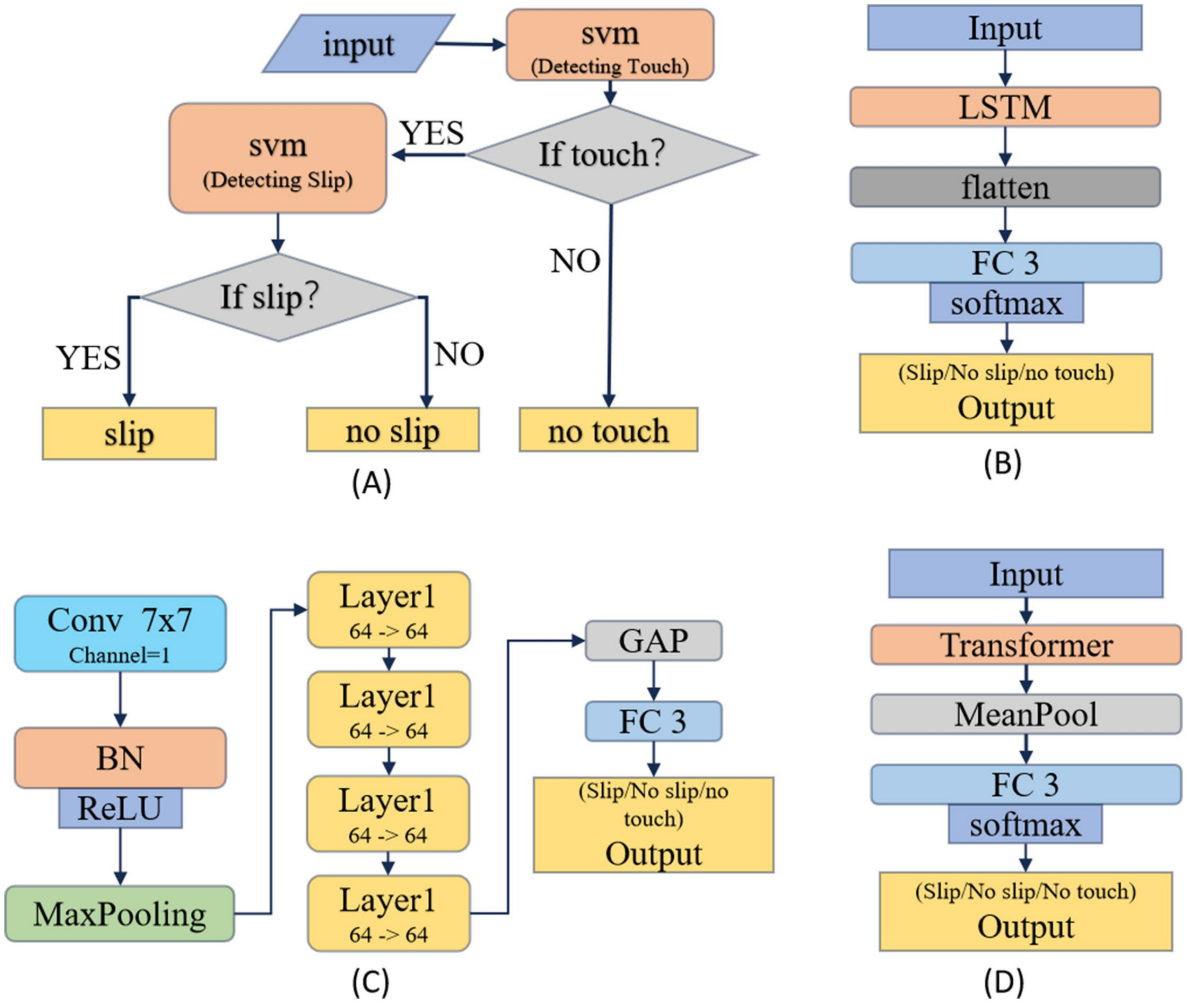
Four classical architectures for slip detection models: **(A)** based on SVM model, **(B)** based on LSTM model, **(C)** based on ResNet model, **(D)** based on Transformer model.

Based on the SVM (Cortes and Vapnik, 1995) method: As shown in the Figure 6A, two support vector machines were trained to achieve the three classifications of “no-touch” “no-slip” and “slip.” Because both “no slip” and “slip” indicate contact with an object, these two categories belong to “touch.” Therefore, the first support vector machine is used to recognize whether there is contact (“touch”), the second support vector machine detects sliding within the “touch” category.

Based on the LSTM (Hochreiter and Schmidhuber, 1997) model, as shown in Figure 6B: Inputting a 16×15 array, it passes through an LSTM network, a flattening layer, two fully connected layers, and finally outputs the probabilities of belonging to each category. The hidden layer dimension and the number of recurrent neural network layers in the LSTM network are both set to 10. During testing, we observed that increasing the size of the LSTM network initially increased the classification accuracy, but then decreased. The best performance was achieved when the hidden layer dimension and the number of recurrent neural network layers reached 10. However, when the number of layers reached 50, the accuracy dropped to 56%.

Based on the ResNet18 (He et al., 2016) model, as shown in Figure 6C: Compared to the standard ResNet18, the number of input channels in the first convolutional layer has been reduced from three to one, and the output dimensions of the final fully connected layer have been adjusted from 1,000 to 3 to match the classification task. The rest of the architecture remains unchanged.

Based on the Transformer (Vaswani et al., 2017) model, as shown in Figure 6D: The input to the model is a 16×15 array. An average pooling layer and a fully connected layer are added after the Transformer. The best performance is achieved when both the encoder and decoder consisting of a single layer.

**Figure 7:**
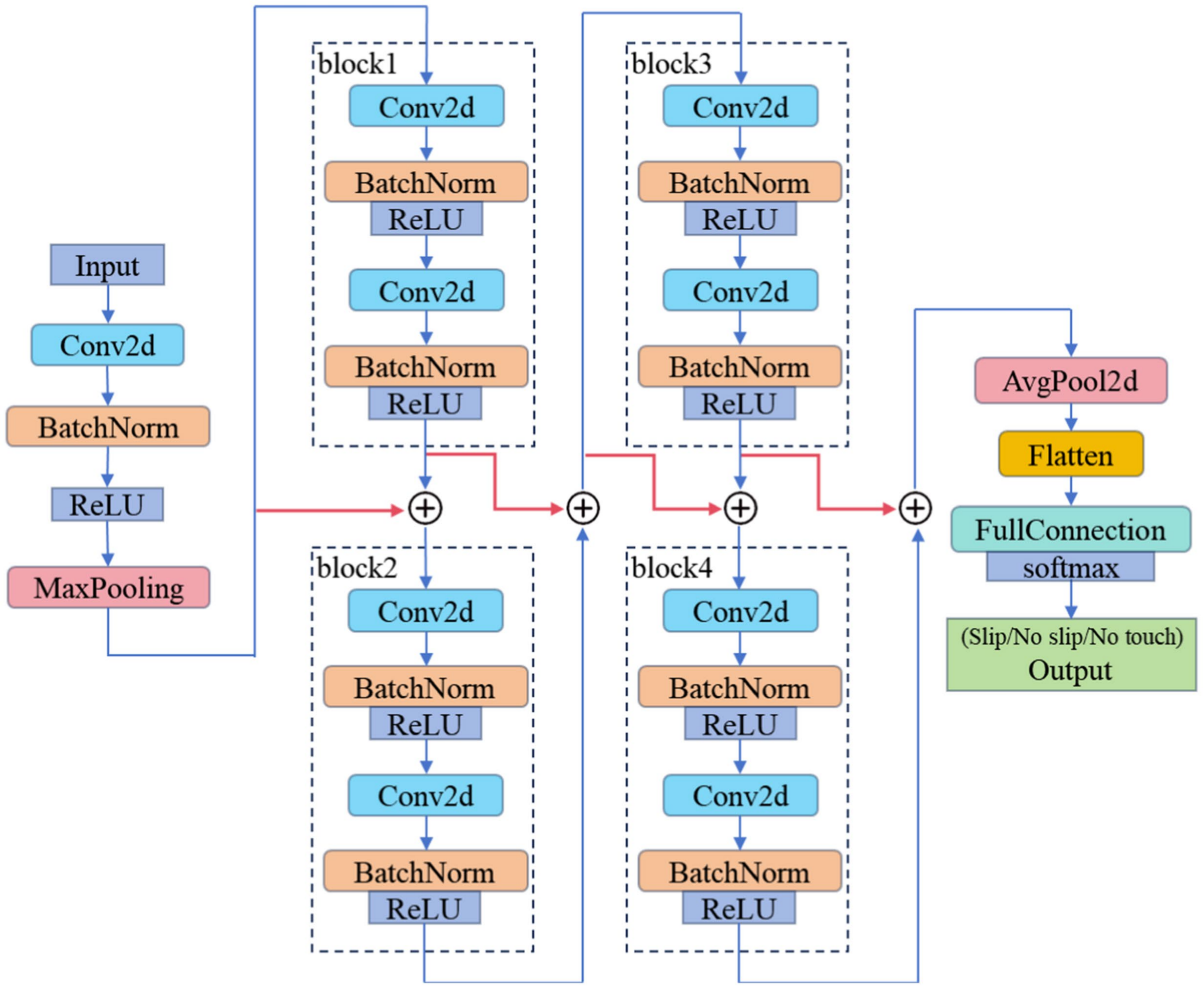
USD ConvNet-DG: universal slip detection convolutional network for different grasp types.

Although ResNet18 achieved over 99% accuracy on the validation set, its accuracy just reached 70% on the test set, which is unacceptable for practical applications. ResNet18 has over 10 million parameters, which does not match the scale of our training dataset. Therefore, as shown in Figure 7, we designed USDConvNet-DG based on the design principles of ResNet18:

1. Residual connections: these connections help mitigate the vanishing gradient problem in deep networks, allowing more efficient gradient flow and facilitating the training of deeper architectures.
2. Hierarchical feature extraction: ResNet18 employs a progressively deeper hierarchical structure, extracting features from lower to higher levels through multiple convolutional layers. Similarly, USDConvNet-DG adopts a block-based design, where each block consists of multiple convolutional operations, enabling finer feature extraction while enhancing the network’s representation capacity.
3. Batch normalization (BN): USDConvNet-DG retains BN layers after each convolution, standardizing data distribution to accelerate convergence, reduce the risk of gradient vanishing, and stabilize the training process for slip detection.
4. Multi-scale feature integration: ResNet18 integrates multi-scale features through residual blocks and layer-wise feature extraction. USDConvNet-DG combines multi-layer convolution and residual connections to effectively extract multi-scale features across different grasp types and contact states, improving performance in slip detection tasks.

The network takes a 2D input, which is processed by a series of convolutional layers. The first layer is a Conv2d (2D convolution) followed by Batch Normalization and a ReLU activation function being followed by MaxPooling, which reduces the spatial dimensions of the feature map. After multiple tests, we found that four blocks are the most suitable. Each block consists of two convolutional layers (Conv2d) with Batch Normalization. The blocks represent different levels of feature extraction with increasing depth, and contributing to a more complex and rich feature representation. The feature map is then flattened and passed through a fully connected layer (FullConnection), which helps in classification. The final layer outputs one of the three categories: slip, no-slip, or no-touch. USDConvNet-DG achieved a maximum accuracy of 97% on the test set.

### 3.7 Training

Furthermore, all tactile sensing, slip detection networks, and robotic five-finger hand control algorithms are executed on a PC equipped with an Intel Core i7-12700K processor (3.60 GHz, 12 cores, 20 threads) and an NVIDIA RTX 3080 Ti GPU. The codes are implemented using PyTorch and Python, running on the Windows 11 operating system.

## 4 Results

This section primarily discusses related test results based on different methods. The result is based on four trained grasping gestures. Overall, the recognition accuracy for the “no-touch” state is higher than other two categories. The classification performance of the method based on USDConvNet-DG is the best, while the performance of SVM method is poorest.

The performance of different models as shown in Table 3, which provides a detailed comparison in terms of accuracy on the validation dataset (grasp types A, B, C, D) and the test dataset (grasp types E and F), prediction time, and the number of parameters. We selected the highest accuracy from the 24 epochs, then averaged and calculated the standard deviation of the ten accuracy values. Given the high demand for real-time performance in slip detection, we also tested the prediction time of various methods.

**Table 3:** Quantitative comparison of different methods

Model	Accuracy		Prediction time	Parameters
	Validation dataset	Test dataset		
SVM	62.81 ± 1.03%	51.34 ± 3.24%	0.08 ms	<100
LSTM	91.24 ± 1.40%	65.69 ± 4.85%	1.30 ms	10,163
	63.38%	42.85%	12.26 ms	10,630,403
Transformer	96.09 ± 0.89%	68.38 ± 2.35%	1.57 ms	129,499
	63.33%	42.76%	129.55 ms	11,034,156
ResNet	99.67 ± 0.06%	78.35 ± 5.27%	2.66 ms	11,171,779
	99.84 ± 0.06%	75.10 ± 5.50%	2.12 ms	709,155
	99.14 ± 0.20%	72.71 ± 6.72%	1.66 ms	47,499
	95.75 ± 0.67%	77.38 ± 10.54%	1.23 ms	2,305
ResNet18 + FFT + Filter	99.02 ± 0.11%	97.09 ± 1.40%	2.66 ms	11,171,779
USDConvNet-DG	97.07 ± 0.18%	86.46 ± 9.58%	1.26 ms	2,395
USDConvNet-DG + FFT	97.78 ± 0.20%	96.65 ± 1.34%	1.26 ms	2,395
USDConvNet-DG + Filter	97.02 ± 0.32%	89.62 ± 4.25%	1.26 ms	2,395
USDConvNet-DG + FFT + Filter	97.71 ± 0.29%	97.12 ± 1.08%	1.26 ms	2,395

The SVM-based classification method had the shortest prediction time, only 0.08 ms, but with low accuracy. When the number of parameters reaches the scale of 10 million, the LSTM and Transformer models achieve approximately 63% accuracy on the validation dataset and 43% on the test dataset, which is about 30% lower than the accuracy of ResNet18. Particularly, the prediction time of the Transformer exceeds 129.55 ms, which is unacceptable for real-time tasks. Additionally, both LSTM and Transformer exhibit slow convergence. The original LSTM lacks residual connections, so multiple LSTM layers can lead to gradient vanishing issues, making it difficult to converge. Moreover, slip detection primarily focuses on local changes in force tactile data, such as short-term high-frequency features. While the self-attention mechanism of the Transformer is applied to capture global long-range dependencies, this capability may not align well with the requirements of slip detection tasks. The complexity of the Transformer may introduce unnecessary computational overhead, whereas convolutional networks are more straightforward and effective for this application.

The ResNet-based classification method has very high accuracy on the validation dataset, but its prediction time is the longest, with over 10 million parameters, making its scale too large to be conveniently integrated into a robotic hand. Thus, we attempted to decrease the number of parameters for ResNet network. We found that the accuracy on the validation dataset decreased by less than 1% when the parameter exceeded 40 k. However, reducing the parameters further resulted in a more pronounced decrease, with accuracy dropping by more than 5%. Specifically, when the parameters are reduced to approximately 2 k, the accuracy on the training dataset decreased by around 4%, but the accuracy on the test dataset improve to 77.38%. These findings suggest that a smaller parameter count may enhance generalization on the test dataset, though it slightly compromises performance on the training and validation datasets. This phenomenon is known as “DEEP DOUBLE DESCENT,” which is common in ResNet and convolutional networks (Nakkiran et al., 2021).

The combination of FFT and filtering with USDConvNet-DG yields the best overall results, with over 97% accuracy on validation dataset and test dataset. These results provide stronger evidence that the network demonstrates robust generalization across diverse grasp types, not limited to the initially trained or tested categories. This model maintains a short prediction time (1.26 ms) and the same low number of parameters (2,395), making it the most effective and efficient model among those tested.

**Figure 8:**
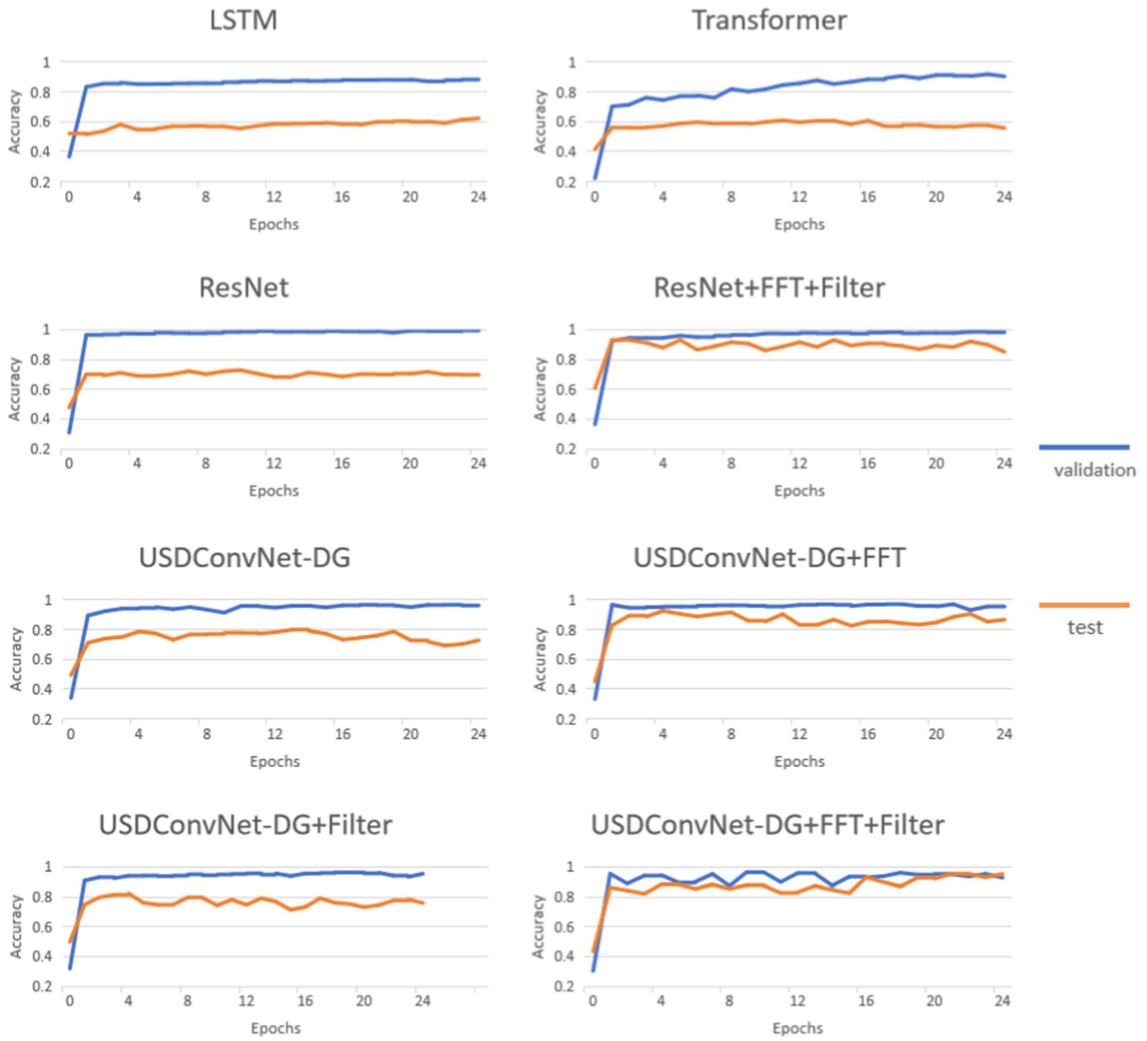
Performance of learning-based method during training.

Figure 8 presents the performance of different models on the validation and test dataset. The epoch was set to 24. The six methods are tested ten times, and the accuracy of each epoch was averaged to capture the overall trend. The following observations can be made:

The accuracy of all models is higher on the validation set than on the test set, and exceeds 90%. For validation, LSTM performs worst, and the accuracy of the Transformer gradually increases to around 90% as the number of epochs increases. However, both models only achieve about 60% accuracy on the test dataset, showing weak generalization in this problem and struggling to generalize well to untrained grasp types. ResNet shows the highest accuracy on the validation set, and its accuracy on the test set is about 10% higher. The USDConvNet-DG we propose performs slightly lower than ResNet on the validation set, but it outperforms ResNet on the test set. When applying FFT and filtering to ResNet, the validation accuracy remains consistently high, and the test accuracy improves compared to using ResNet alone.

When FFT is used to preprocess the training data, the test accuracy of USDConvNet-DG improves significantly. The improvement is relatively smaller with filtered data. Overall, combining FFT and filtering with USDConvNet-DG results in the most stable and high accuracy on the test dataset, closely approaching the validation accuracy. This model appears to effectively balance feature extraction and generalization, making it the most robust among the tested configurations.

It is worth exploring whether the data collected from different grasp types contributes differently to universal slip detection. Therefore, we designed a controlled experiment as follows: five of the six grasp types were used to train the model, and the remaining one was used to test the model to obtain the accuracy. The test results for the six grasp types are shown in Table 4. The accuracy is lower when Type A is not included in the training set, indicating that Type A contributes more to universal slip detection.

**Table 4:** The model’s accuracy on test datasets with different grasp types.

Model	Accuracy on single dataset					
	Type A	Type B	Type C	Type D	Type E	Type F
LSTM	56.73%	67.73%	65.35%	68.28%	66.31%	64.39%
Transformer	60.89%	69.20%	66.16%	73.91%	70.57%	69.84%
ResNet18	70.98%	84.24%	81.93%	77.02%	78.63%	80.55%
ResNet18 + FFT + Filter	88.90%	96.07%	97.58%	98.13%	97.87%	99.05%
USDConvNet-DG	76.31%	89.47%	87.72%	88.53%	87.41%	88.06%
USDConvNet-DG + FFT	89.17%	96.26%	97.53%	98.27%	97.97%	98.61%
USDConvNet-DG + Filter	77.89%	86.57%	91.23%	89.02%	88.91%	87.20%
USDConvNet-DG + FFT + Filter	89.15%	97.82%	99.15%	98.24%	97.01%	99.84%

Table 1 shows the accuracy of USDConvNet-DG with varying numbers of grasp types. The test dataset consists of Grasp Types E and F, and the number of epochs is set to 20. As the grasp type is 1, the model was trained separately on the four training sets (A, B, C, D). The test was repeated five times. Finally, the average and standard deviation of the 20 accuracy results were calculated. When the grasp type is 2, the training dataset is a combination of two grasp types. The model was trained separately on the six training sets (AB, AC, AD, BC, BD, CD), and the accuracy improved significantly. When the grasp type is 3, the training dataset consists of three grasp types. When the grasp type is 4, all four grasp types together form a single training dataset, and the improvement in accuracy is minimal. Overall, with the number of grasp types increases, the accuracy on the test dataset improves.

To test the effectiveness of recognizing tactile events locally (i.e., per fingertip), we trained USDConvNet-DG using individual sensor data (3 × 16 arrays). Each fingertip was independently detected whether slippage occurred. If any one of the five fingertips detected slippage, the system classified the event as slippage; otherwise, it was classified as no slippage. The results showed that the model’s accuracy decreased to 93.48% on the training dataset and 80.58% on the test dataset. Additionally, the computation time increased to 5.34 ms because the detection process was repeated five times to evaluate the tactile events for all five fingertips individually. These findings indicate that considering all five fingertips as a whole is more effective than recognizing tactile events locally. Treating the five fingertips as a unified system not only improves the model’s accuracy but also reduces computational overhead.

We applied 5-fold cross-validation to measure the accuracy for all six types, where the datasets for all six grasp types and the no-touch state were randomly and evenly divided into six groups. One group was used as the test set, while the other five groups were used for training and validation. The test was repeated five times. The accuracy on the validation set is 97.60%, with a standard deviation of 1.06%. The accuracy on the test set is 97.15%, with a standard deviation of 1.05%. These results provide evidence that the network demonstrates robust generalization across diverse grasp types.

Overall, the USDConvNet-DG model combined with FFT and filtering demonstrates the best generalization on the test set while maintaining high validation accuracy and short computing time, suggesting that this configuration is the most effective for slip detection in this experiment.

Moreover, we designed two groups of physical experiments to test the accuracy and real-time performance of USDConvNet-DG in real-world scenarios. In one group, the grasp state was detected in real-time while external force was applied to the object. In the other group, the grasping force was increased (from 100mN to 700mN) upon slip detection, demonstrating that the force adjustment could be completed with the object slipping by less than 1 cm. However, there were still limitations in accurately detecting minimal contact and slight slippage. For instance, slight slippage around the 6-s mark in Video 1 was not detected, and the contact state was misclassified in Video 3 due to minimal contact. Additionally, a clear delay existed between the end of slip and switching back to the no-slip state, as robotic hand re-established a stable state after detecting slippage. Video demonstrations are available at https://github.com/sunshine486/show.

## 5 Conclusion

Overall, this work presented a novel framework, USDFrame-DG, that performs slip detection across different grasp types for a five-fingered robotic hand equipped with integrated 3-axis force sensors. The proposed framework achieved this by utilizing a large dataset of various grasp types to train models, enabling it to detect slip across a wide range of untrained grasp types. It is found that the accuracy on the test gradually improve as the number of grasp types in the training set increased. To identify the most suitable network for universal slip detection, we designed three deep networks based on three classic deep learning models. Then, a lightweight network called USDConvNet-DG was designed based on the structure of the best-performing ResNet18. It has fewer parameters, shorter computation time, and no significant drop in accuracy. Using FFT and a digital high-pass filter for data preprocessing facilitated the extraction of spectral features and reduced low-frequency noise, significantly improving recognition accuracy. Physical experiments were conducted to demonstrate that the proposed framework can quickly detect the state of a grasp and adjust grasp force in real-time. These experiments also demonstrated that the ability to detect slip serves as a useful and reliable metric for determining grasp stability. Future research will focus on three aspects: First, we will explore the implementation of our framework on robotic hands with varying numbers of fingers and a diverse range of sensors. Second, the framework can be applied to adjust grasp strategies to achieve grasp stabilization. Third, a robotic hand equipped with slip detection should be capable of grasping unknown objects using minimal force while preventing them from slipping or being dropped.

## Data Availability

The raw data supporting the conclusions of this article will be made available by the authors, without undue reservation.
